# Supplementary material for: Identification of photoperiod-induced specific miRNAs in the adrenal glands of Sunite sheep (Ovis aries)
Source: Front Vet Sci. 2022 Jul 22;9:888207. doi: 10.3389/fvets.2022.888207 (PMC9354845; doi:10.3389/fvets.2022.888207)
Supplement: Supplementary file 1 [file Data_Sheet_1.ZIP › Supplementary file/miRDeep2 file.zip!/miRDeep2 file/Know_Structure/LP42A1/oar-mir-27a.pdf]

miRBase precursor : oar-mir-27a
Total read count : 300185
oar-miR-27a read count : 299933
remaining reads : 252

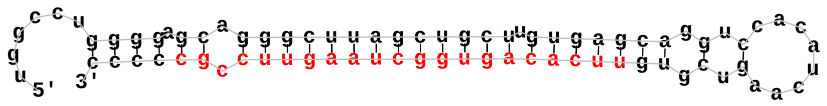

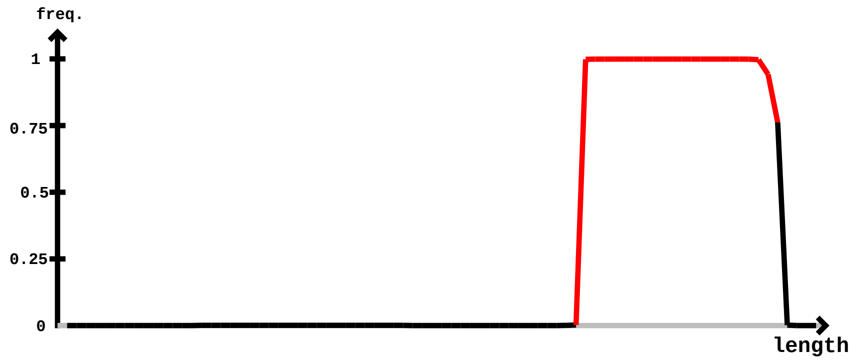

oar-miR-27a

5'- uggccuggggagcagggcuuagcugcuugugagcagguccacaucaagucguguucacaguggcuaaguuccgccccc -3'	exp		
......((((.((.(((((((((((((.(((((((.(.(........).).))))))))))))))))))))).))))))	reads	mm	sample
.............agggcuuagcugcuugug...	2	0	seq
.............agggcuuagcugcuugugagc..	32	0	seq
.............agggcuuagcugcuugugagca...	208	0	seq
.............agggcuuagcugcuugugagcagg...	6	0	seq
.............agggcuuagcugcuugugagcaggu..	4	0	seq
....uguucacaguggcuaaguuc.......	9	0	seq
....uguucacaguggcuaaguucc......	11	0	seq
....uguucacaguggcuaaguucU......	16	1	seq
....uguucacaguggcuaaguuccg.....	140	0	seq
....uguucacaguggcuaaguucUg.....	2	1	seq
....uguucacaguggcuaaguuccgc....	25	0	seq
....uguucacaguggcuaaguuccgcc...	3	0	seq
....Uuucacaguggcuaaguu........	1	1	seq
....guucacaguggcuaaguuc.......	1	0	seq
....guucacaguggcuaaguucc......	4	0	seq
....guucacaguggcuaaguucU......	24	1	seq
....guucacaguggcuaaguucUg.....	73	1	seq
....guucacaguggcuaaguuccg.....	2	0	seq
....guucacaguggcuaaguuccgc....	4	0	seq
....guucacaguggcuaaguucUgc....	336	1	seq
....uucacaguggcuaaguu........	54	0	seq
....uucacaguAgcuaaguuc.......	1	1	seq
....uucacaguggcuaaguuc.......	638	0	seq
....uucacaguggcuaaguuG.......	4	1	seq
....uucacaguggcuaaguucc......	2904	0	seq
....uucacaguggcuaaguucU......	13384	1	seq
....uucacaguggcuaaguuccg.....	23738	0	seq
....uucacaguggcuaaguuccA.....	6497	1	seq
....uucacaguggcuaaguucUg.....	23517	1	seq
....uucacaguggcuaaguucUgc....	221316	1	seq
....uucacaguggcuaaguuccgc....	6444	0	seq
....uucacaguggcuaaguuccgcc...	517	0	seq
....uucacaguggcuaaguuccgccc..	8	0	seq
....ucacaguggcuaaguuc.......	1	0	seq
....ucacaguggcuaaguucc......	2	0	seq

oar-miR-27a

uggccuggggagcagggcuuagcugcuugugagcagguccacaucaagucguguucacaguggcuaaguuccgccccc			
....ucacaguggcuaaguucU......	17	1	seq
....ucacaguggcuaaguuccg.....	21	0	seq
....ucacaguggcuaaguuccA.....	4	1	seq
....ucacaguggcuaaguucUg.....	16	1	seq
....ucacaguggcuaaguuccgc....	7	0	seq
....ucacaguggcuaaguucUgc....	158	1	seq
....ucacaguggcuaaguuccgcc...	1	0	seq
....cacaguggcuaaguucU......	2	1	seq
....cacaguggcuaaguucUg.....	2	1	seq
....cacaguggcuaaguuccg.....	2	0	seq
....cacaguggcuaaguucUgc....	23	1	seq
....acaguggcuaaguucUg.....	2	1	seq
....acaguggcuaaguuccg.....	1	0	seq
....acaguggcuaaguucUgc....	1	1	seq
